# Supplementary material for: A median fin derived from the lateral plate mesoderm and the origin of paired fins
Source: Nature. 2023 May 24;618(7965):543–9. doi: 10.1038/s41586-023-06100-w (PMC10266977; doi:10.1038/s41586-023-06100-w)
Supplement: Supplementary file 1 — Reporting Summary [file 41586_2023_6100_MOESM1_ESM.pdf]

nature portfolio

Corresponding author(s): Tom J. Carney

Last updated by author(s): Apr 9, 2023

Reporting Summary

Nature Portfolio wishes to improve the reproducibility of the work that we publish. This form provides structure for consistency and transparency in reporting. For further information on Nature Portfolio policies, see our Editorial Policies and the Editorial Policy Checklist.

Statistics

For all statistical analyses, confirm that the following items are present in the figure legend, table legend, main text, or Methods section.

n/a	Confirmed	
☐	☒	The exact sample size (*n*) for each experimental group/condition, given as a discrete number and unit of measurement
☐	☒	A statement on whether measurements were taken from distinct samples or whether the same sample was measured repeatedly
☐	☒	The statistical test(s) used AND whether they are one- or two-sided<br>*Only common tests should be described solely by name; describe more complex techniques in the Methods section.*
☒	☐	A description of all covariates tested
☒	☐	A description of any assumptions or corrections, such as tests of normality and adjustment for multiple comparisons
☐	☒	A full description of the statistical parameters including central tendency (e.g. means) or other basic estimates (e.g. regression coefficient) AND variation (e.g. standard deviation) or associated estimates of uncertainty (e.g. confidence intervals)
☐	☒	For null hypothesis testing, the test statistic (e.g. *F*, *t*, *r*) with confidence intervals, effect sizes, degrees of freedom and *P* value noted<br>*Give P values as exact values whenever suitable.*
☒	☐	For Bayesian analysis, information on the choice of priors and Markov chain Monte Carlo settings
☒	☐	For hierarchical and complex designs, identification of the appropriate level for tests and full reporting of outcomes
☒	☐	Estimates of effect sizes (e.g. Cohen's *d*, Pearson's *r*), indicating how they were calculated

Our web collection on statistics for biologists contains articles on many of the points above.

Software and code

Policy information about availability of computer code

Data collection	Zeiss ZEN Blue 3.6<br>Fiji ImageJ ver. 1.52p<br>IMARIS 9.9.1 (Oxford Instruments)<br>Avizo 3D software (ThermoFisher Scientific)
Data analysis	GraphPad Prism 9

For manuscripts utilizing custom algorithms or software that are central to the research but not yet described in published literature, software must be made available to editors and reviewers. We strongly encourage code deposition in a community repository (e.g. GitHub). See the Nature Portfolio guidelines for submitting code & software for further information.

Data

Policy information about availability of data

All manuscripts must include a data availability statement. This statement should provide the following information, where applicable:

- Accession codes, unique identifiers, or web links for publicly available datasets
- A description of any restrictions on data availability
- For clinical datasets or third party data, please ensure that the statement adheres to our policy

The authors confirm that all relevant data are provided in the results section of this paper, in its Extended Data files and in the Source Data file. The data for

measurements of fin size and cell number in Extended Data Figure 3m-o are also available in Figshare with the identifier https://doi.org/10.6084/m9.figshare.22269769.

Human research participants

Policy information about studies involving human research participants and Sex and Gender in Research.

Reporting on sex and gender	N/A
Population characteristics	N/A
Recruitment	N/A
Ethics oversight	N/A

Note that full information on the approval of the study protocol must also be provided in the manuscript.

Field-specific reporting

Please select the one below that is the best fit for your research. If you are not sure, read the appropriate sections before making your selection.

☐ Life sciences ☐ Behavioural & social sciences ☒ Ecological, evolutionary & environmental sciences

For a reference copy of the document with all sections, see nature.com/documents/nr-reporting-summary-flat.pdf

Ecological, evolutionary & environmental sciences study design

All studies must disclose on these points even when the disclosure is negative.

Study description	Expression analysis of tissue developmental origin across multiple species
Research sample	Danio rerio (WT strain: AB)<br>Oryzias latipes (WT strain: Cab)<br>Polyodon spathula<br>Xenopus tropicalis<br>Xenopus laevis<br>Petromyzon marinus<br>Carassius auratus (Ranchu strain)
Sampling strategy	n>=5 for all statistical analyses on fin size counts. All expression experiments were performed multiple times on different animal batches
Data collection	Data collected by microscopy
Timing and spatial scale	Developmentally relevant timepoints chosen
Data exclusions	Nil
Reproducibility	All expression experiments were performed multiple times on different animal batches. Findings were consistent across different repeat experiments
Randomization	Embryos selected at random for imaging
Blinding	NA

Did the study involve field work? ☐ Yes ☒ No

Reporting for specific materials, systems and methods

We require information from authors about some types of materials, experimental systems and methods used in many studies. Here, indicate whether each material, system or method listed is relevant to your study. If you are not sure if a list item applies to your research, read the appropriate section before selecting a response.

Materials & experimental systems

n/a	Involved in the study
☐	☒ Antibodies
☒	☐ Eukaryotic cell lines
☒	☐ Palaeontology and archaeology
☐	☒ Animals and other organisms
☒	☐ Clinical data
☒	☐ Dual use research of concern

Methods

n/a	Involved in the study
☒	☐ ChIP-seq
☒	☐ Flow cytometry
☒	☐ MRI-based neuroimaging

Antibodies

Antibodies used	anti-Col2A1 (II-II6B3, DSHB)<br>anti-EGFP (TP401, Torrey Pines)<br>zns-5 (AB_10013796, ZIRC)<br>anti-SM22 alpha/Transgelin (ab14106, Abcam)<br>Alexa Fluor-488 Donkey anti-rabbit, Invitrogen, A21206<br>Alexa Fluor-647 Donkey anti-rabbit, Invitrogen, A31573<br>Alexa Fluor-546 Donkey anti-mouse, Invitrogen, A10036<br>Alexa Fluor-647 Donkey anti-mouse, Invitrogen, A31571
Validation	See validation and associated references on the antibody supplier websites:<br>https://dshb.biology.uiowa.edu/II-II6B3<br>http://chemokine.com/Houston/rat&other/GFP.PDF<br>https://zfin.org/ZDB-ATB-081002-37<br>https://www.abcam.com/taglntransgelin-antibody-ab14106.html<br>https://www.thermofisher.com/antibody/secondary/query

Animals and other research organisms

Policy information about studies involving animals; ARRIVE guidelines recommended for reporting animal research, and Sex and Gender in Research

Laboratory animals	Danio rerio (WT strain: AB)<br>Oryzias latipes (WT strain: Cab)<br>Polyodon spathula<br>Xenopus tropicalis<br>Xenopus laevis<br>Petromyzon marinus<br>Carassius auratus (Ranchu strain)
Wild animals	No wild animals were used in the study.
Reporting on sex	Most experiments were conducted during larval stages before sexual dimorphism occurs. Otherwise gender is reported for adults (in Extended Data Figure 9)
Field-collected samples	No field collected samples were used in the study
Ethics oversight	IMCB, A*STAR, Singapore (IACUC #140924)<br>Nanyang Technological University (IACUC #A18002)<br>CU Anschutz Medical Campus (protocol number 979)<br>National University of Singapore (IACUC #BR19-0120 and #BR22-1497)<br>James Madison University (IACUC #20-1601)<br>California Institute of Technology (IACUC #1436)<br>University of Manchester and the Home Office (PFDA14F2D)<br>Monash Animal Ethics Committee under license (#30347)

Note that full information on the approval of the study protocol must also be provided in the manuscript.
